# Supplementary material for: Gut Microbiota and Non-Alcoholic Fatty Liver Disease Severity in Type 2 Diabetes Patients
Source: J Pers Med. 2021 Mar 23;11(3):238. doi: 10.3390/jpm11030238 (PMC8004607; doi:10.3390/jpm11030238)
Supplement: Supplementary file 1 [file jpm-11-00238-s001.pdf]

Table S1. Target sequences of the materials utilized in the study

Target	Primer (5'-3')
Phylum: *Firmicutes*	F: GGAGYATGTGGTTTAATTCGAAGCA
	R: AGCTGACGACAACCATGCAC
C. leptum group (IV)	F: GTTGACAAAACGGAGGAAGG
	R: GACGGGCGGTGTGTACAA
Faecalibacterium prausnitzii	F: AGATGGCCTCGCGTCCGA
	R: CCGAAGACCTTCTTCCTCC
Phylum: *Bacteroidetes*	F: GGARCATGTGGTTTAATTCGATGAT
	R: AGCTGACGACAACCATGCAG
Bacteroides	F: GTCAGTTGTGAAAGTTTGC
	R: CAATCGGGAGTTCTTCGTG
Phylum: *Actinobacteria*	
Bifidobacterium	F: AGGGTTCGATTCTGCTCAG
	R: CATCCGGCATTACCACCC
Phylum: *Proteobacteria*	
Escherichia coli	F: CATGCCGCGTGTATGAAGAA
	R: CGGGTAACGTCAATGAGCAAA
Phylum:*Verrucomicrobia*	
Akkermansia muciniphila	F:CAGCACGTGAAGGTGGGGAC
	R:CCTTGCGGTTGGCTTCAGAT
